# A Conserved Arginine-Rich Motif within the Hypervariable N-Domain of *Drosophila* Centromeric Histone H3 (CenH3^CID^) Mediates BubR1 Recruitment

**DOI:** 10.1371/journal.pone.0013747

**Published:** 2010-10-29

**Authors:** Mònica Torras-Llort, Sònia Medina-Giró, Olga Moreno-Moreno, Fernando Azorín

**Affiliations:** Institute of Molecular Biology of Barcelona, CSIC, and Institute for Research in Biomedicine, IRB Barcelona, Barcelona, Spain; Virginia Tech, United States of America

## Abstract

**Background:**

Centromere identity is determined epigenetically by deposition of CenH3, a centromere-specific histone H3 variant that dictates kinetochore assembly. The molecular basis of the contribution of CenH3 to centromere/kinetochore functions is, however, incompletely understood, as its interactions with the rest of centromere/kinetochore components remain largely uncharacterised at the molecular/structural level.

**Principal Findings:**

Here, we report on the contribution of *Drosophila* CenH3^CID^ to recruitment of BubR1, a conserved kinetochore protein that is a core component of the spindle attachment checkpoint (SAC). This interaction is mediated by the N-terminal domain of CenH3^CID^ (NCenH3^CID^), as tethering NCenH3^CID^ to an ectopic reporter construct results in BubR1 recruitment and BubR1-dependent silencing of the reporter gene. Here, we also show that this interaction depends on a short arginine (R)-rich motif and that, most remarkably, it appears to be evolutionarily conserved, as tethering constructs carrying the highly divergent NCenH3 of budding yeast and human also induce silencing of the reporter. Interestingly, though NCenH3 shows an exceedingly low degree of conservation, the presence of R-rich motives is a common feature of NCenH3 from distant species. Finally, our results also indicate that two other conserved sequence motives within NCenH3^CID^ might also be involved in interactions with kinetochore components.

**Conclusions:**

These results unveil an unexpected contribution of the hypervariable N-domain of CenH3 to recruitment of kinetochore components, identifying simple R-rich motives within it as evolutionary conserved structural determinants involved in BubR1 recruitment.

## Introduction

Centromere function ensures accurate chromosome segregation during mitosis and meiosis, as the centromere dictates assembly of the kinetochore that, in turn, regulates the spindle attachment checkpoint (SAC), which delays anaphase onset until all chromosomes are correctly attached in a bipolar fashion to the mitotic spindle. Centromere identity is regulated epigenetically by deposition of the centromere-specific histone H3 variant CenH3 that, being exclusively found at centromeres, constitutes the structural and functional foundation for kinetochore assembly and function [1], [2], [3], [4], [5], [6], [7], [8]. CenH3 is essential for viability, being required for centromeric localisation of all centromere/kinetochore proteins analysed to date.

Little is known, however, about the actual molecular/structural basis of the contribution(s) of CenH3 to kinetochore assembly and function, as its interactions with the rest of centromere/kinetochore proteins is just beginning to be understood. In this context, it was recently reported that CENP-N and CENP-C, which are components of the constitutive centromere associated network (CCAN) in vertebrates [9], [10], directly interact with human CenH3^CENP-A^-containing nucleosomes *in vitro* [11], [12]. These interactions involve the C-terminal and centromere targeting (CATD) domains of CenH3^CENP-A^, respectively. Here, we report on the contribution of the N-terminal domain of *Drosophila* CenH3^CID^ (NCenH3^CID^) to recruitment of BubR1, an evolutionarily conserved kinetochore protein that is a core component of the spindle attachment checkpoint (SAC) [13], [14], [15]. This interaction is mediated by a simple arginine (R)-rich motif within the hypervariable NCenH3^CID^ domain. Our results also suggest that this interaction is likely conserved in the highly divergent NCenH3 of budding yeast and humans. Most remarkably, though NCenH3 is poorly conserved through evolution [16], [17], the presence of R-rich motives is a common feature of NCenH3 from distant species, including budding yeast and humans [2]. In *Drosophila*, NCenH3^CID^ contains two other conserved motives that might also mediate interactions with kinetochore components. Altogether, these results indicate that conserved sequence motives within the hypervariable NCenH3 domain mediate centromere/kinetochore interactions.

## Results and Discussion

### Targeting NCenH3^CID^ to an ectopic *white*-reporter construct silences reporter expression

**Figure 1:**
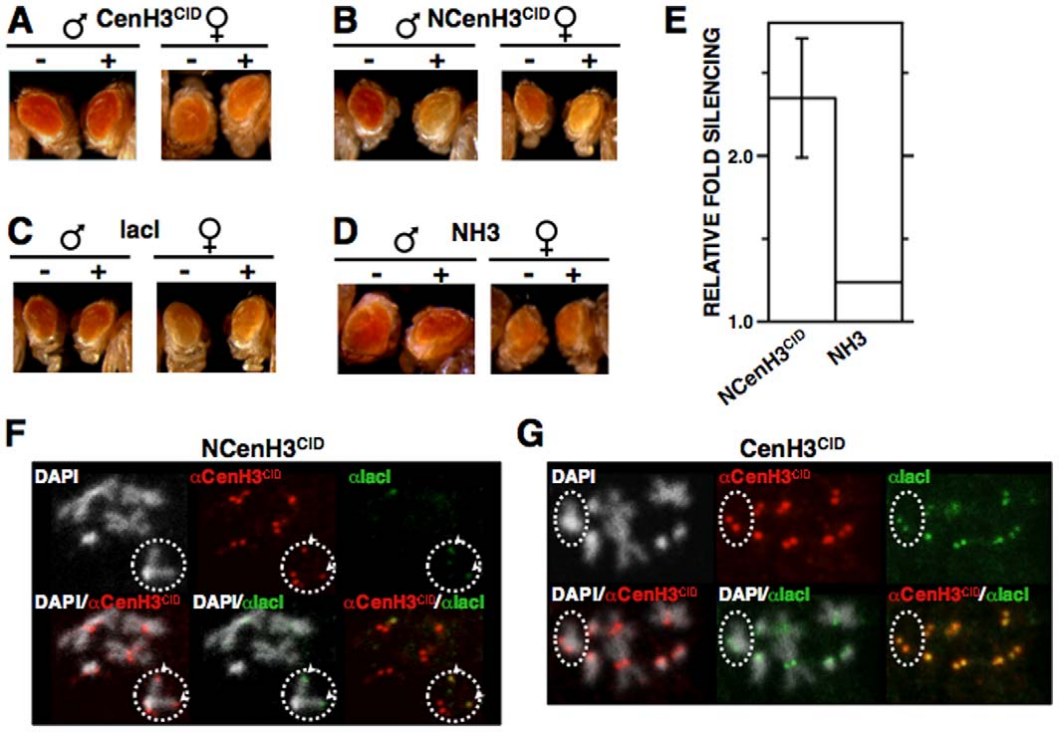
Tethering NCenH3^CID^ to a *white-*reporter silences reporter expression. (A–D) The eye phenotype of *S9.2* flies expressing the indicated lacI-fused proteins (+) is compared to that of siblings where no fused protein is expressed (−). Results are presented for both female and male individuals. (E) Quantitative analysis is presented for lines expressing NCenH3^CID^-lacI and NH3^CID^-lacI constructs. Relative fold silencing is expressed as the ratio between OD_480_ of control S9.2 lines expressing no fused protein and that of lines expressing the indicated constructs. For NCenH3^CID^-lacI, results correspond to the average of three independent lines. For NH3^CID^-lacI, results are presented for a single representative line. (F and G) NCenH3^CID^-lacI, but not CenH3^CID^-lacI, bind to the ectopic *white*-reporter construct. Fused proteins were expressed in *157.1* flies, where the *white-*reporter is inserted at a distal position on the X-chromosome, and localisation was determined in mitotic chromosomes by immunostaining with αlacI (green) and αCenH3^CID^ (red), which also detects endogenous CenH3^CID^ at centromeres. Dotted circles indicate X-chromosomes. Arrows indicate co-localisation of αCenH3^CID^ and αlacI signals at ectopic sites on the X-chromosome, reflecting binding of NCenH3^CID^-lacI to the reporter. DNA was stained with DAPI. See Figure S4 for a description of the constructs.

To analyse the contribution of CenH3^CID^ to the regulation of centromere/kinetochore interactions, we performed ectopic targeting experiments using *Drosophila* transgenic lines carrying a *white*-reporter transgene that contains multiple binding sites for the bacterial lacI repressor at the regulatory region, about 500 bp upstream from the reporter gene. In these experiments, lines *S9.2* and *157.1* were used, which contain 46 and 256 lacI-repeats inserted on the third- and X-chromosome, respectively [18]. We anticipated that, if resulting in recruitment of kinetochore proteins, tethering of fused CenH3^CID^-lacI proteins would interfere with expression of the *white*-reporter gene, which is easily monitored by analysing changes in eye pigmentation. As shown in Figure 1A, in line *S9.2*, expression of a CenH3^CID^-lacI fusion does not significantly affect *white* expression. In contrast, expression of an NCenH3^CID^-lacI fused protein, which carries only the N-terminal domain of CenH3^CID^, significantly silences reporter expression (Figure 1B and 1E). This effect is specific of NCenH3^CID^, as no silencing is observed in flies expressing the lacI-DNA-binding domain alone (Figure 1C) [18], [19], or, more important, an NH3-lacI construct (Figure 1D and 1E), carrying the N-terminal domain of canonical histone H3. Similar results were obtained when expression was carried out in line *157.1* (Figure S1A, D and E), though, in this case, the observed effects are weaker since expression of the *white*-reporter is low in control flies expressing no fused proteins [18], [19]. Immunolocalisation experiments showed that CenH3^CID^-lacI fails to localise to the reporter construct but, on the contrary, incorporates to all centromeres (Figures 1G and S2A), indicating that, in CenH3^CID^-lacI, the histone-fold domain (HFD) of CenH3^CID^ predominates over the lacI-DNA-binding domain, so that CenH3^CID^-lacI incorporates into nucleosomes, like endogenous CenH3^CID^ does, being specifically deposited at centromeres. On the other hand, NCenH3^CID^-lacI exclusively localises to the ectopic reporter sites (Figure 1F and S2B). From these results, we conclude that lack of silencing observed in the case of CenH3^CID^-lacI is actually the consequence of its failure to localise to the ectopic reporter sites.

### Tethering NCenH3^CID^-lacI results in ectopic BubR1 recruitment

**Figure 2:**
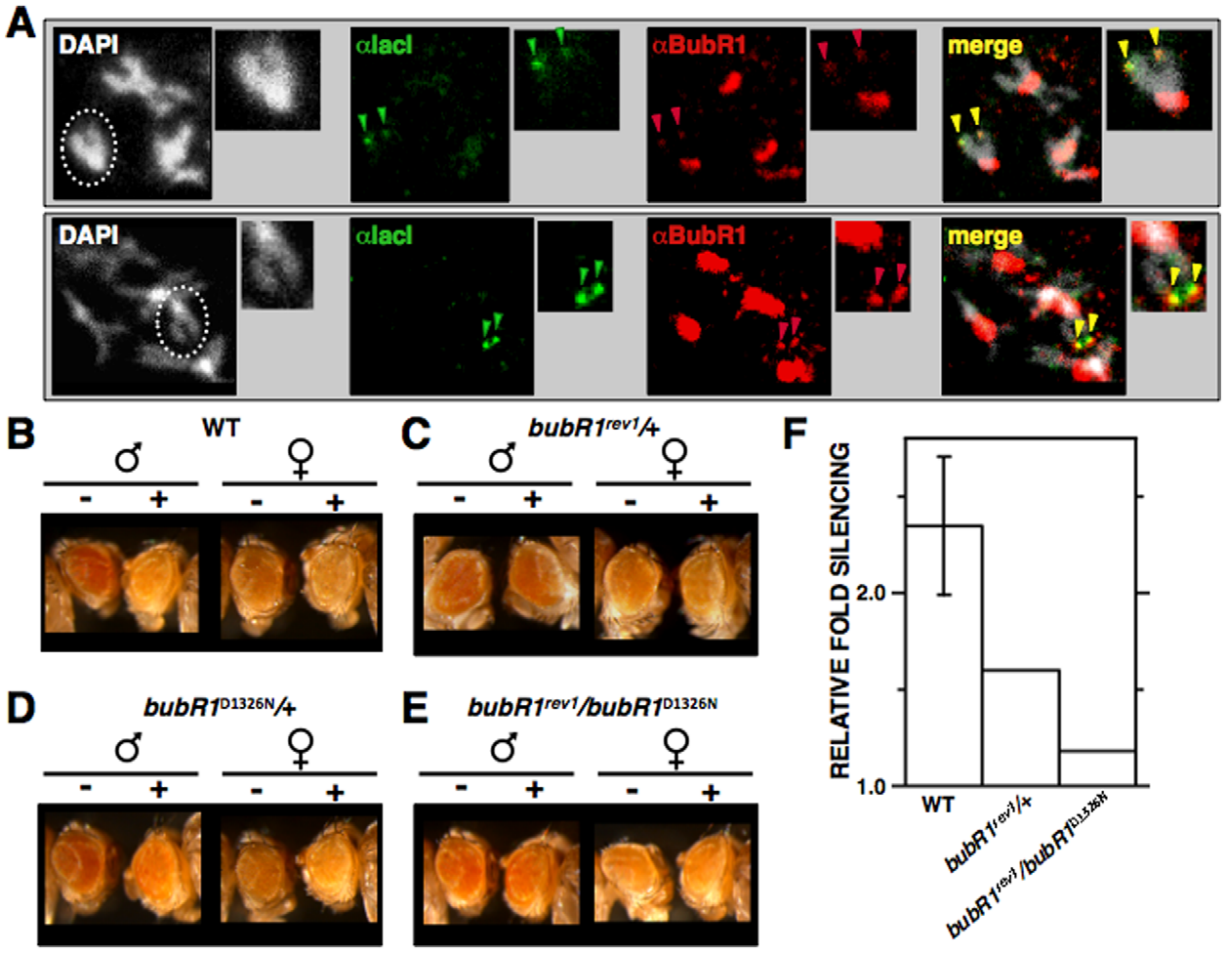
NCenH3^CID^ mediates BubR1 recruitment. (A) Localisation of NCenH3^CID^-lacI and BubR1 was determined in mitotic chromosomes from *157.1* flies by immunostaining with αlacI (green) and αBubR1 (red). Dotted circles indicate X-chromosomes. Arrows indicate co-localisation of αlacI and αBubR1 signals at ectopic sites on the X-chromosome, reflecting recruitment of BubR1 by NCenH3^CID^-lacI. Enlarged images are shown on the right of each panel for easier visualisation. DNA was stained with DAPI. Two independent examples are presented. On the bottom, pictures were recorded to a higher intensity to better visualise the ectopic αBubR1 signals observed on the X-chromosome. (B–E) Silencing induced by NCenH3^CID^-lacI depends on BubR1. The eye phenotype of *S9.2* flies expressing NCenH3^CID^-lacI (+) or not (−) is presented in the indicated genetic backgrounds. Results are presented for both female and male individuals. (F) Quantitative analysis is presented for flies expressing NCenH3^CID^-lacI in the indicated genetic backgrounds. Relative fold silencing is expressed as the ratio between OD_480_ of control S9.2 flies expressing no fused protein and that of flies expressing NCenH3^CID^-lacI in the indicated genetic backgrounds. For wild-type, results correspond to the average of three independent lines. For mutants, results are presented for a single representative line.

**Figure 3:**
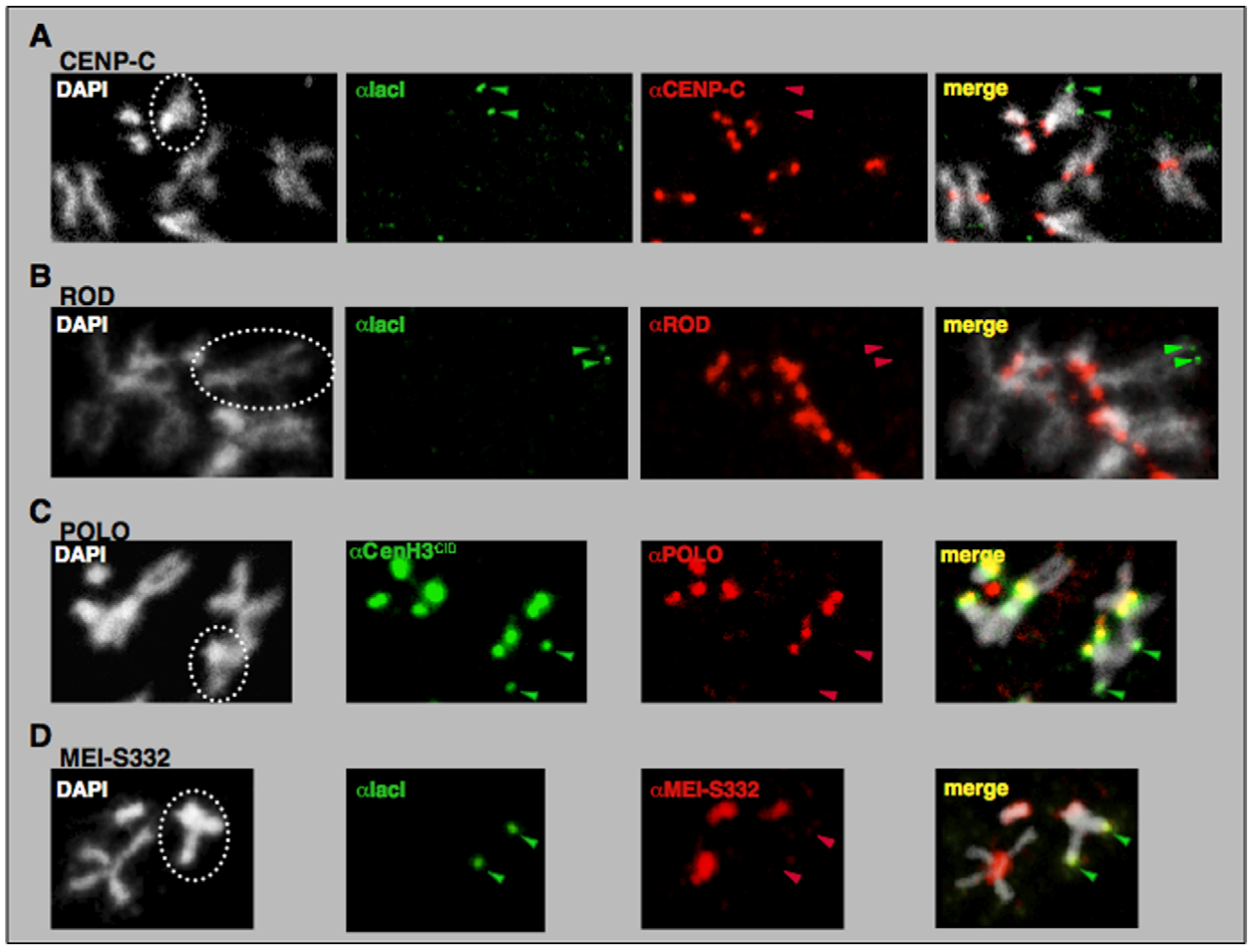
Targeting of NCenH3^CID^-lacI to the reporter construct in *157.1* flies does not results in ectopic recruitment of CENP-C. (A), ROD (B), POLO (C) or MEI-S332 (D). Localisation of NCenH3^CID^-lacI (green) and CENP-C, ROD, POLO or MEI-S332 (red) was determined in mitotic chromosomes by immunostaining with the indicated specific antibodies. Dotted circles indicate X-chromosomes. Arrows indicate ectopic αlacI or αCenH3^CID^ signals on the X-chromosome, which reflect binding of NCenH3^CID^-lacI to the reporter construct.

Next, we addressed whether ectopic targeting of NCenH3^CID^-lacI actually results in recruitment of kinetochore proteins. For this purpose, immunolocalisation experiments using antibodies against several *Drosophila* kinetochore proteins were performed (Figures 2 and 3). In mitotic chromosomes, tethering NCenH3^CID^-lacI to the reporter construct results in recruitment of BubR1, as distinct ectopic αBubR1 signals are detected on the X-chromosome in approximately 15% of chromosomes (N = 60; p<0.001) (Figure 2A). These αBubR1 signals overlap with αlacI signals, which reflect binding of NCenH3^CID^-lacI to the lacI-repeats and, therefore, mark the position corresponding to the reporter. Ectopic αBubR1 signals are weak compared to those observed at the kinetochore, indicating that ectopic BubR1 recruitment is less efficient than at the kinetochore, which might simply reflect the limited number of NCenH3^CID^-lacI molecules that can be targeted to the reporter, a maximum of 256 copies. It is also possible that additional factors are involved in stabilising BubR1 at the kinetochore. Actually, as discussed below (see “General considerations and implications”), recruitment and maintenance of BubR1 at kinetochores might involve different mechanisms. In good agreement with these results, silencing of the reporter depends on BubR1, as it is suppressed by *bubR1* mutations (Figure 2B–F). In these experiments, *bubR1^rev1^* and *bubR1^D1326N^* mutants were used, which correspond to a deletion and a point-mutation at the catalytic kinase-domain, respectively [20], [21]. In heterozygous *bubR1^rev1^*/+ flies, silencing induced by NCenH3^CID^-lacI is strongly suppressed (Figure 2C and 2F), when compared to control wild-type flies (Figure 2B and 2F). This dominant suppressor effect is observed in approximately 50% of the off-spring (N = 75), the rest showing only slight or no suppression. *bubR1^rev1^* mutation is homozygous lethal, so that silencing induced by NCenH3^CID^-lacI could not be analysed in homozygous *bubR1^rev1^*/*bubR1^rev1^* flies. However, suppression is enhanced in trans-heterozygous *bubR1^rev1^*/*bubR1^D1326N^*, where the complete off-spring (N = 55) shows strong suppression (Figure 2E and 2F). On the other hand, heterozygous *bubR1^D1326N^*/+ flies show only slight suppression (Figure 2D).

Results described above indicate that, in *Drosophila*, NCenH3^CID^ is involved in recruitment of BubR1, an evolutionarily conserved kinetochore protein, which is a core component of the spindle attachment checkpoint (SAC). Current models for SAC function suggest that unattached kinetochores recruit SAC components, such as BubR1, to generate a diffusible signal that delays anaphase onset. Therefore, ectopic recruitment of BubR1 could reflect formation of a functional ectopic kinetochore. This possibility, however, is highly unlikely since targeting NCenH3^CID^ does not result in recruitment of other essential kinetochore components, including CENP-C [22], ROD [23], [24], POLO [25], and MEI-S332/Sgo [26], [27] (Figure 3). Also in agreement with this hypothesis, ectopic targeting of NCenH3^CID^ does not induce any detectable proliferation defects (not shown), which is contrary to what would be expected if resulting in efficient formation of an ectopic kinetochore [28]. Altogether, these results argue against formation of a functional kinetochore, indicating that NCenH3^CID^ is not sufficient by itself to support kinetochore assembly. Actually, it was recently reported that, in vertebrates, both the C-terminal and central CATD domains of CenH3^CENP-A^ mediate interactions with two essential CCAN-components, CENP-C and CENP-N [11], [12], being, therefore, required for full kinetochore assembly.

### Silencing induced by CenH3^CID^ depends on a simple arginine-rich sequence motif

**Figure 4:**
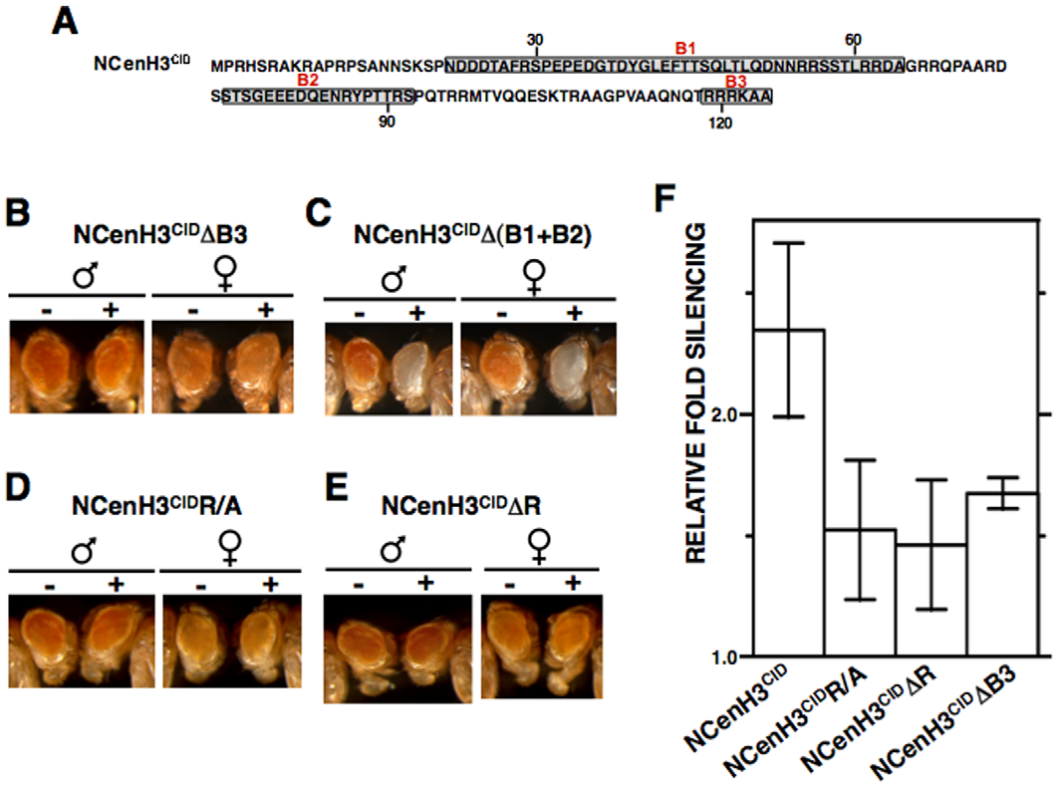
Silencing induced by NCenH3^CID^ depends on a conserved simple arginine (R)-rich motif. (A) Amino acid sequence of the N-terminal domain of *D. melanogaster* CenH3^CID^. Sequence motives (B1, B2 and B3) that are conserved amongst distant *Drosophila* species are indicated. (B–E) The eye phenotype of *S9.2* flies expressing the indicated NCenH3^CID^-lacI deletions (+) is compared to that of siblings where no fused protein is expressed (−). Results are presented for both female and male individuals. (F) Quantitative analysis is presented for lines expressing the indicated constructs. Relative fold silencing is expressed as the ratio between OD_480_ of control S9.2 lines expressing no fused protein and that of lines expressing each construct. Results correspond to the average of four (NCenH3^CID^R/A), three (NCenH3^CID^), and two (NCenH3^CID^ΔR and NCenH3^CID^ΔB3) independent lines. See Figure S4 for a description of the constructs.

**Figure 5:**
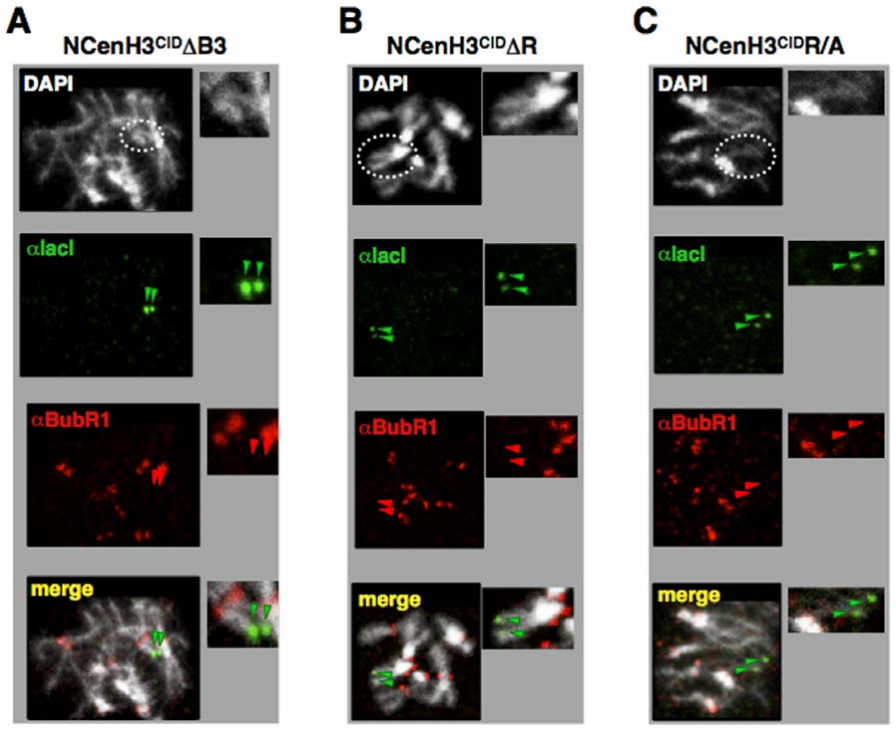
R-residues within B3 motif mediate recruitment of BubR1. Co-localisation of BubR1 with NCenH3^CID^ΔB3-lacI (A), NCenH3^CID^ΔR-lacI (B) and NCenH3^CID^R/A-lacI (C) was determined in mitotic chromosomes from *157.1* flies by immunostaining with αlacI (green) and αBubR1 (red). Dotted circles indicate X-chromosomes. Arrows indicate the position of the reporter construct on the X-chromosome. Enlarged images are shown on the right of each panel for easier visualisation. DNA was stained with DAPI.

Next, we asked about the molecular basis of the contribution of NCenH3^CID^ to BubR1 recruitment. Within the *Drosophila* genus, NCenH3^CID^ shows significant variability. However, sequence comparison of NCenH3^CID^ from a broad group of *Drosophila* species [17], allowed identification of three sequence motives (B1, B2 and B3) (Figure 4A) that, being evolutionarily conserved over 25 million years, were good candidates to mediate recruitment of BubR1. To test this possibility, we performed deletion analyses, where the contribution of each conserved motif to silencing of the *white*-reporter was determined in ectopic targeting experiments. As shown in Figure 4, motif B3 has a major contribution, as its deletion strongly impairs silencing (Figure 4B and 4F) and is capable by itself to induce robust silencing of the reporter (Figure 4C). Moreover, deletion of motives B1 or B2 has no detectable effect on reporter silencing (Figure S3B and S3C). Motif B3 corresponds to a rather simple sequence, ^119^RRRKAA^124^, showing a peculiar enrichment in arginine (R) residues. As a matter of fact, silencing induced by NCenH3^CID^ is strongly impaired when R-residues within B3 are replaced by alanine (A) (Figure 4D and 4F) or deleted (Figure 4E and 4F), and, concomitant to the lack of silencing, no ectopic αBubR1 signals are detected in these cases (Figure 5). Altogether, these results identify R-residues within B3 as involved in BubR1 recruitment.

### Highly divergent NCenH3 of budding yeast and human also silence reporter expression

**Figure 6:**
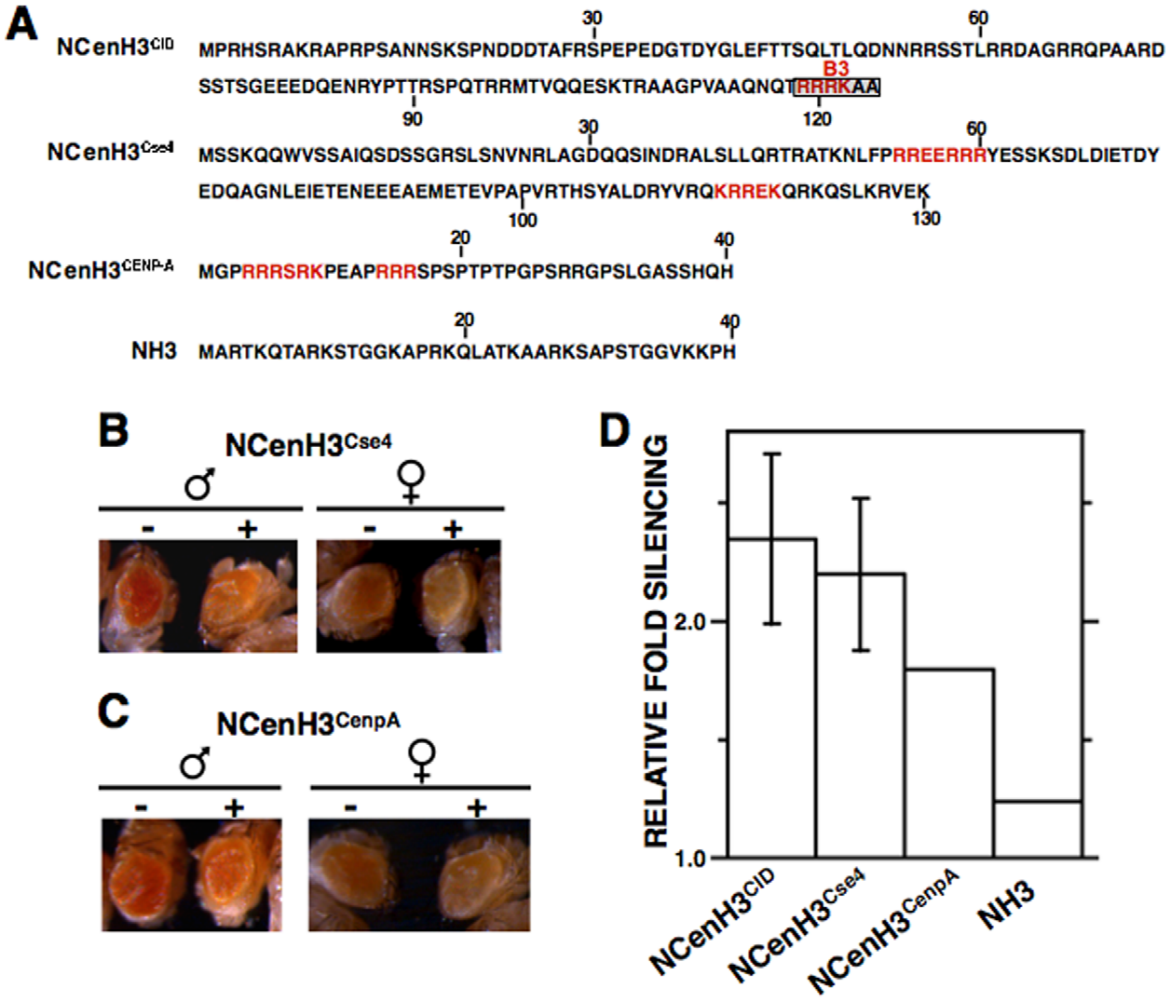
Expression of fused proteins carrying highly divergent NCenH3 of budding yeast (NCenH3^Cse4^) and human (NCenH3^CENP-A^) also silence reporter expression. (A) Amino acid sequence of the N-terminal domain of *D. melanogaster* CenH3^CID^, S. *cerevisiae* CenH3^Cse4^, human CenH3^CENP-A^, and canonical histone H3 (NH3). Motif B3 that is responsible for silencing induced by *D. melanogaster* NCenH3^CID^ is indicated. R-rich motives (basic sequences containing ≥40% R) are highlighted in red. (B and C) The eye phenotype of *S9.2* flies expressing the indicated lacI-fused proteins (+) is compared to that of siblings where no fused protein is expressed (−). Results are presented for both female and male individuals. (F) Quantitative analysis is presented for lines expressing the indicated constructs. Relative fold silencing is expressed as the ratio between OD_480_ of control S9.2 lines expressing no fused protein and that of lines expressing each construct. For NCenH3^CID^-lacI and NCenH3^Cse4^-lacI, results correspond to the average of three independent lines. For NCenH3^CENP-A^-lacI, where only 50% of the lines show significant silencing, and NH3-lacI, results are presented for a single representative line. See Figure S4 for a description of the constructs.

In comparison to canonical histone H3, CenH3 is much less well conserved [16], [17]. Homology, that at the histone-fold domain ranges from 40% to 60% identity, is, however, insignificant for NCenH3 that, showing strong variability both in size (ranging from 20 to 200 aa) and sequence, cannot be aligned across different eukaryotic lineages. Therefore, in this context, we asked whether the effects described above are restricted to *Drosophila* CenH3^CID^. To address this question, lacI-fusions carrying NCenH3 from *Saccharomyces cerevisiae* (NCenH3^Cse4^) or humans (NCenH3^CenpA^) were expressed in *Drosophila S9.2* reporter flies and the extent to which they induce silencing of the reporter determined. Both constructs induce silencing (Figure 6B–D), though it is less robust than that observed for NCenH3^CID^, being significant only in a fraction of lines analysed. In the case of NCenH3^Cse4^, 75% (N = 4) of lines show strong silencing similar to that observed for NCenH3^CID^ (Figure 6B and 6D). On the other hand, 50% (N = 4) of lines expressing NCenH3^CenpA^-lacI show significant silencing, which is slightly weaker than that induced by NCenH3^CID^-lacI (Figure 6C and 6D). Similar results were obtained when expression was performed in *157.1* flies (Figure S1B and C). These results strongly suggest that the contribution of NCenH3 to recruitment of BubR1 is conserved through evolution, from *S. cerevisiae* to humans, which is in contrast to its low degree of conservation. It must be noted, however, that a common feature of NCenH3 from distant species is its enrichment in R-residues in comparison to canonical histone H3. As a matter of fact, R-rich motives similar to motif B3 that mediates BubR1 recruitment in *Drosophila*, are present at NCenH3 from most species, including those analysed here, but absent in canonical histone H3 [2] (Figure 6A).

### General considerations and implications

Current models for kinetochore assembly and function suggest that presence of CenH3 at the centromere results in a specialised chromatin structure, which provides a physical foundation to build the kinetochore. CenH3-kinetochore interactions remain, however, incompletely understood at the molecular level. Results reported here identify simple R-rich motives within the hypervariable NCenH3^CID^ as evolutionary conserved structural determinants involved in BubR1 recruitment. Whether the contribution of NCenH3^CID^ to BubR1 recruitment is direct or mediated by additional unidentified factor(s) is uncertain, as GST-pull down assays failed to detect any direct physical interaction between BubR1 and NCenH3^CID^ *in vitro* or upon co-transfection into cultured S2-cells (not shown).

These results, which are based on ectopic targeting experiments, are likely relevant in the context of the endogenous *locus*. BubR1 recruitment is detected in early prometaphase, when kinetochores are bound by only few or no microtubules [29]. Our results suggest that BubR1 recruitment does not require full kinetochore assembly, as ectopic targeting of NCenH3^CID^, which does not result in formation of a functional kinetochore, is capable of recruiting BubR1. Several observations support this hypothesis. On one hand, in chicken DT40 cells, strong reduction of CenH3^CENP-A^ to levels that severely impair centromeric localisation of several CCAN-components (CENP-C, -H, and -I), as well as some outer kinetochore proteins (Nuf2/Hec1, Mad2, and CENP-E), shows only a moderate effect on the initial recruitment of BubR1 in early prometaphase [30]. Furthermore, in *Drosophila*, *cid* mutants, that fail to assemble the kinetochore, show a BubR1-dependent early mitotic delay [31]. Altogether, these observations suggest a BubR1-CenH3 interaction occurring early in mitosis, prior to full kinetochore assembly. Whether this interaction is mediated by NCenH3 remains, however, to be determined. On the other hand, association of BubR1 to metaphase kinetochores appears to depend strongly on kinetochore assembly, as it is strongly destabilised in CenH3^CENP-A^-depleted DT40 cells [30], suggesting that initial recruitment and maintenance of BubR1 at kinetochores involve different mechanisms and, perhaps, fulfil different functions. Actually, BubR1 is known to play multiple roles during mitosis [32].

**Figure 7:**
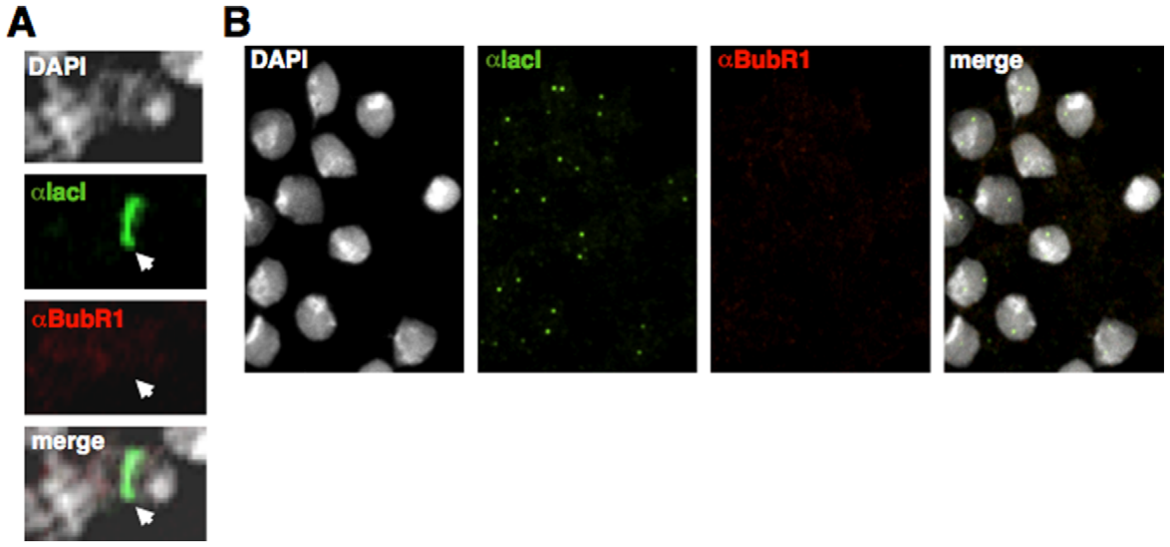
Ectopic targeting of NCenH3^CID^ does not induce BubR1 recruitment in interphase. Localisation of NCenH3^CID^-lacI and BubR1 was determined in polytene chromosomes (A) and in interphase cells from brain squashes (B) by immunostaining with αlacI (green) and αBubR1 (red). αlacI signals indicate binding of NCenH3^CID^-lacI to the reporter construct. DNA was stained with DAPI.

NCenH3^CID^-BubR1 interaction appears to be regulated during cell-cycle progression, as no ectopic αBubR1 signals are detected on polytene chromosomes, which constitute a special type of interphase chromatin, or in interphase nuclei from larval neuroblasts (Figure 7), indicating that, like at the kinetochore, ectopic recruitment of BubR1 by NCenH3^CID^ is constrained to mitosis. In contrast, our results show that NCenH3^CID^-mediated silencing of the *white*-reporter depends on BubR1, indicating that BubR1 is required to repress reporter expression at interphase. BubR1 recruitment at mitosis might stabilise binding of factor(s) required for repression at interphase. It is also possible that BubR1 facilitates chromatin modification, as recent results show that Bub1, a closely related SAC-kinase that plays partially redundant functions, regulates H2AS121-phosphorylation in fission yeast, which lacks BubR1 [33].

In addition to the B3 R-rich motif, *Drosophila* NCenH3^CID^ contains two other evolutionarily conserved regions (motives B1 and B2) (Figure 4A), which might also mediate centromere/kinetochore interactions. Support for this hypothesis comes from the observation that constructs containing only motif B1 or B2 retain some silencing competence (Figure S3D and S3E). However, in comparison to motif B3, the contribution of motives B1 and B2 to silencing is only minor, as by themselves induce much weaker silencing (Figure S3D and S3E), and their deletion does not significantly affect silencing (Figure S3B and S3C, and Figure 4C). Whether silencing induced by motives B1 and B2 also reflects interaction with kinetochore proteins remains, however, to be determined.

Altogether, these observations favour the hypothesis that some kinetochore proteins bind CenH3-chromatin through the recognition of specific sequence motives within the hypervariable NCenH3 domain, unveiling its essential contribution to CenH3 functionality. Results obtained in *S. cerevisiae* support this hypothesis, as NCenH3^Cse4^ is essential for viability and, moreover, interacts genetically with components of COMA, a kinetochore complex that is functionally related to CCAN and mediates protein-protein interactions with other centromere/kinetochore proteins, including the essential CBF3 complex [34], [35].

## Materials and Methods

### Fly stocks

For targeting experiments, CenH3^CID^, NCenH3^CID^, NH3, NCenH3^Cse4^ and NCenH3^CenpA^ were fused at N-terminus of the lacI-DNA-binding domain (see Figure S4 for a description of the constructs), using plasmid lacI^ST^-Topo-TA and cloned into pNHT4 plasmid [36], where expression is driven by the *hsp70*-promoter. For deletion-analyses, NCenH3^CID^-lacI^ST^-Topo-TA was used as template and the various deletions cloned to vector pNHT4 (Figure S4). Transgenic lines were generated in +;+/+;*ry^506^/ry^506^* flies by standard procedures. All constructs were tested for expression and their ability to target the reporter construct determined in polytene chromosomes (Figure S5). *bubR1^rev1^* and *bubR1^D1326N^* mutants are described elsewhere [20], [21]. Lines *S9.2* or *157.1* are also described elsewhere [18].

### Tethering experiments

For tethering experiments, heterozygous flies carrying the indicated lacI-constructs were crossed to homozygous *S9.2* or *157.1* reporter flies. Crosses were, then, subjected to daily heat-shock treatment for 45 min at 37°C and the eye phenotype of flies expressing the corresponding lacI-fused protein compared to that of siblings, of the same sex and age, expressing no fused protein. For each lacI-construct, at least four independent lines were analysed. When silencing induced by NCenH3^CID^-lacI was analysed in *bubR1^rev1^* and *bubR1^D1326N^* mutant backgrounds, heterozygous NCenH3^CID^-lacI/+ males were crossed to *bubR1^rev1^*/+ or *bubR1^D1326N^*/+ reporter *S9.2* females. When silencing was analysed in trans-heterozygous *bubR1^rev1^*/*bubR1^D1326N^* flies, *bubR1^D1326N^*/+ reporter *S9.2* females were crossed to *bubR1^rev1^*/+ males carrying NCenH3^CID^-lacI. For quantitative analyses, eye pigment was extracted with 30% acid-ethanol (pH = 2) according to [37] and OD_480_ determined in a Nanodrop 1000/3.7. Extraction was performed from 20 heads obtained from male individuals. Relative fold silencing was then expressed as the ratio between OD_480_ of control S9.2 line expressing no fused protein and that of lines expressing the corresponding constructs.

### Immunostaining experiments

For immunostaining experiments, homozygous flies carrying the indicated lacI-constructs were crossed to homozygous *157.1* reporter flies and crosses were subjected to daily heat-shock treatment for 45 min at 37°C to the third-instar larvae stage. After the last heat-shock treatment, larvae were left to recover at 25°C for 2 h prior to dissection. Brains were, then, incubated in 0.5 mg/ml colcemid in PBS for 1.5 h before fixation in 3.7% formaldehyde. Neuroblasts squashes and immunostainings were performed as described elsewhere [19], [38] using rabbit polyclonal αCenH3^CID^ (1∶500), rabbit polyclonal αBubR1 (Rb666) (1∶1000) [39], mouse monoclonal αPolo (mab294) (1∶100) [40], rabbit αCenpC polyclonal antibody (1∶5000) [22], rabbit αRod polyclonal antibody (1∶200) [41], guinea pig αMEI-S332 polyclonal antibody (1∶1000) [27] and mouse monoclonal αlacI (clone 9A5, Upstate) (1∶150). For visualization, slides were mounted in Mowiol (Calbiochem-Novabiochem) containing 0.2 ng/ml DAPI (Sigma) and visualized by confocal microscopy (Leica TCS SP2-AOBS).

## Supporting Information

Figure S1 Tethering NCenH3-lacI to the ectopic white-reporter of *157.1* flies induces silencing of the reporter gene. (A–E) The eye phenotype of flies expressing the indicated fused proteins (+) is compared to that of siblings where no fused protein is expressed (−). Results are presented only for male individuals.
(0.46 MB PDF)

Figure S2 In interphase nuclei, CenH3^CID^-lacI incorporates to centromeres. CenH3^CID^-lacI (A) and NCenH3^CID^-lacI (B) were expressed in *157.1* flies carrying an ectopic white reporter construct inserted at a distal position on the X-chromosome. Localisation of the fused proteins was determined in interphase cells from brain squashes of third instar larvae by immunostaining with αCenH3^CID^ (red) and αlacI (green). In cells expressing CenH3^CID^-lacI (A), all αCenH3^CID^ signals co-localise with αlacI, indicating incorporation of CenH3^CID^-lacI to centromeres. In contrast, in cells expressing NCenH3^CID^-lacI (B), co-localisation is restricted to two-spots (indicated by the arrows), reflecting binding of the fused protein to the ectopic reporter construct.
(0.09 MB PDF)

Figure S3 Motives B1 and B2 retain silencing competence. (A) Amino acid sequence of the N-terminal domain of *D. melanogaster* CenH3^CID^. Conserved sequence motives (B1, B2 and B3) are indicated. (B–E) The eye phenotype of *S9.2* flies expressing the indicated NCenH3^CID^-lacI deletions (+) is compared to that of siblings where no fused protein is expressed (−). Results are presented for both female and male individuals. See Figure S4 for a description of the constructs.
(1.45 MB PDF)

Figure S4 Constructs used in these experiments. Schematic representation of fused proteins used in these experiments. The position of motives B1, B2 and B3 is indicated. Numbers correspond to amino acid positions on the corresponding sequences. DNA-binding domain of lacI is indicated in red.
(0.02 MB PDF)

Figure S5 Constructs used in these experiments target the reporter construct in polytene chromosomes. Localisation of the indicated constructs was determined in polytene chromosomes by immunostaining with αlacI (green). DNA was stained with DAPI.
(0.15 MB PDF)
