# Supplementary figures and images for: A Conserved Arginine-Rich Motif within the Hypervariable N-Domain of Drosophila Centromeric Histone H3 (CenH3CID) Mediates BubR1 Recruitment
Source: PLoS One. 2010 Oct 29;5(10):e13747. doi: 10.1371/journal.pone.0013747 (PMC2966416; doi:10.1371/journal.pone.0013747)

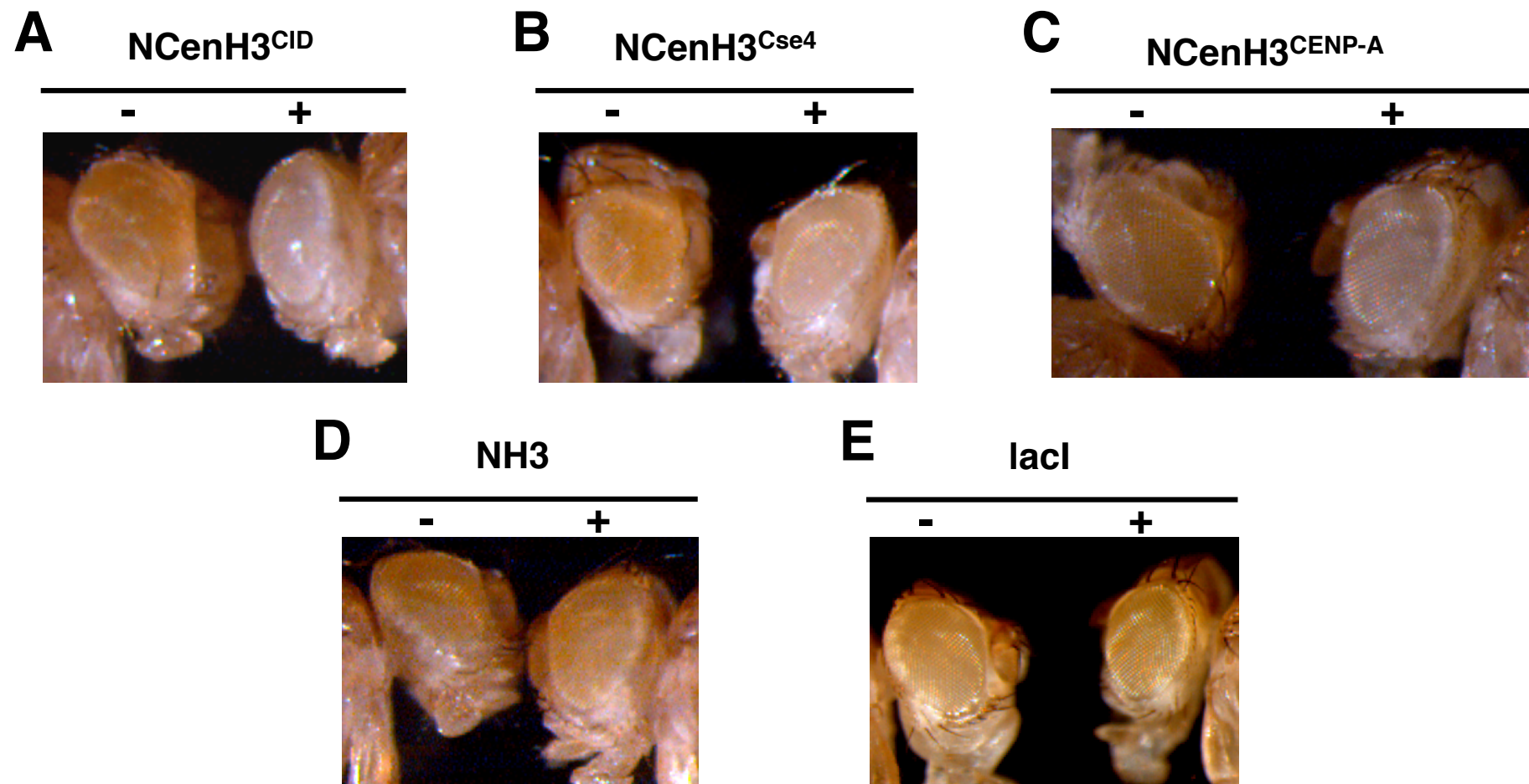

**FIGURE S1**

Supplement: Figure S1 — Tethering NCenH3-lacI to the ectopic white-reporter of 157.1 flies induces silencing of the reporter gene. (A–E) The eye phenotype of flies expressing the indicated fused proteins (+) is compared to that of siblings where no fused protein is expressed (−). Results are presented only for male individuals. (0.46 MB PDF) [file pone.0013747.s001.pdf]

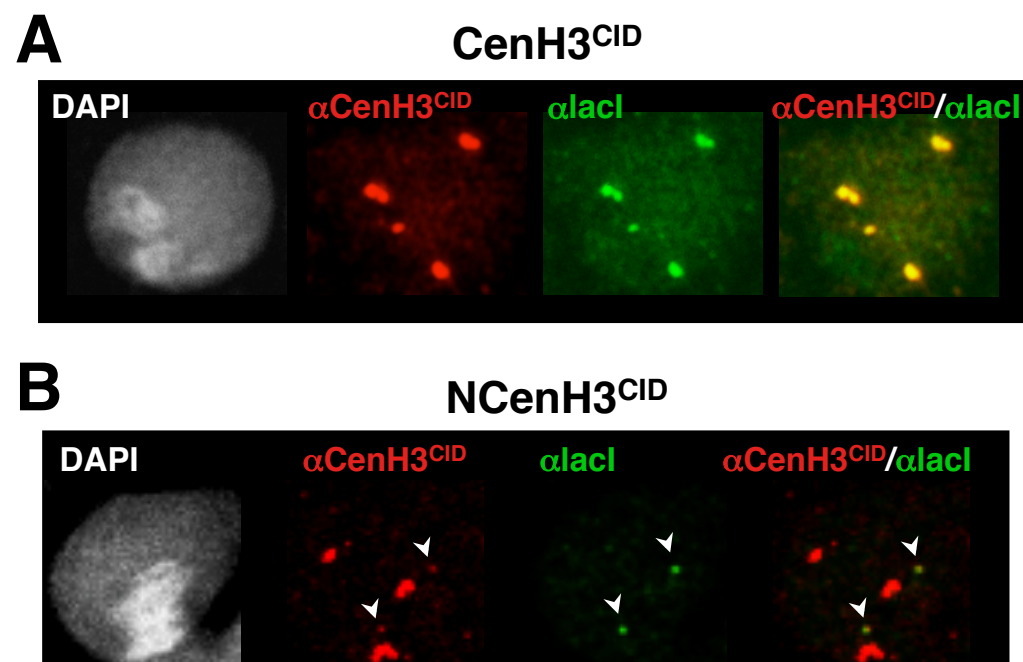

**FIGURE S2**

Supplement: Figure S2 — In interphase nuclei, CenH3CID-lacI incorporates to centromeres. CenH3CID-lacI (A) and NCenH3CID-lacI (B) were expressed in 157.1 flies carrying an ectopic white reporter construct inserted at a distal position on the X-chromosome. Localisation of the fused proteins was determined in interphase cells from brain squashes of third instar larvae by immunostaining with αCenH3CID (red) and αlacI (green). In cells expressing CenH3CID-lacI (A), all αCenH3CID signals co-localise with αlacI, indicating incorporation of CenH3CID-lacI to centromeres. In contrast, in cells expressing NCenH3CID-lacI (B), co-localisation is restricted to two-spots (indicated by the arrows), reflecting binding of the fused protein to the ectopic reporter construct. (0.09 MB PDF) [file pone.0013747.s002.pdf]

**A**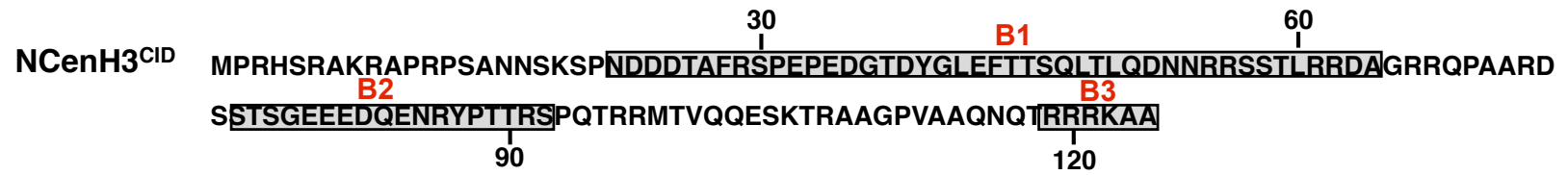**B**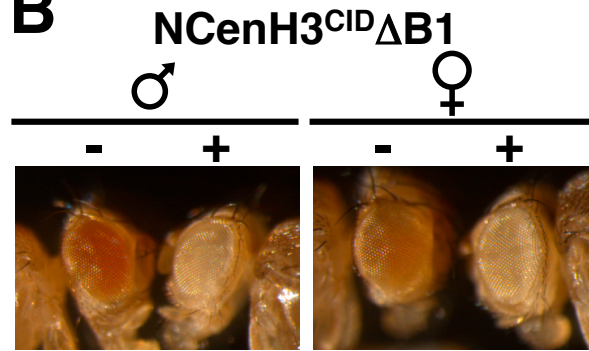**C**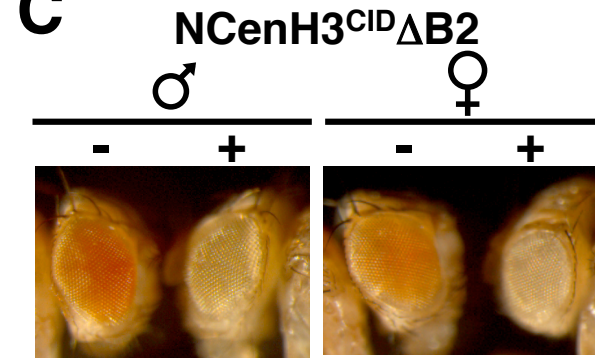**D**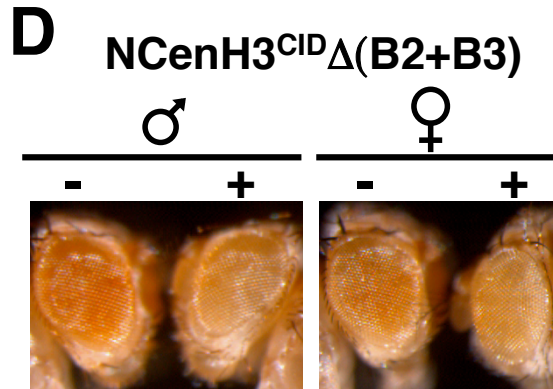**E**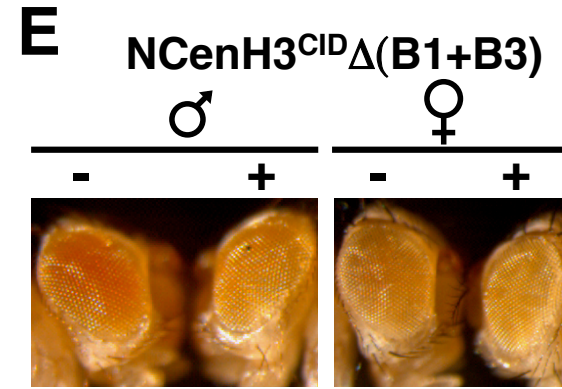**FIGURE S3**

Supplement: Figure S3 — Motives B1 and B2 retain silencing competence. (A) Amino acid sequence of the N-terminal domain of D. melanogaster CenH3CID. Conserved sequence motives (B1, B2 and B3) are indicated. (B–E) The eye phenotype of S9.2 flies expressing the indicated NCenH3CID-lacI deletions (+) is compared to that of siblings where no fused protein is expressed (−). Results are presented for both female and male individuals. See Figure S4 for a description of the constructs. (1.45 MB PDF) [file pone.0013747.s003.pdf]

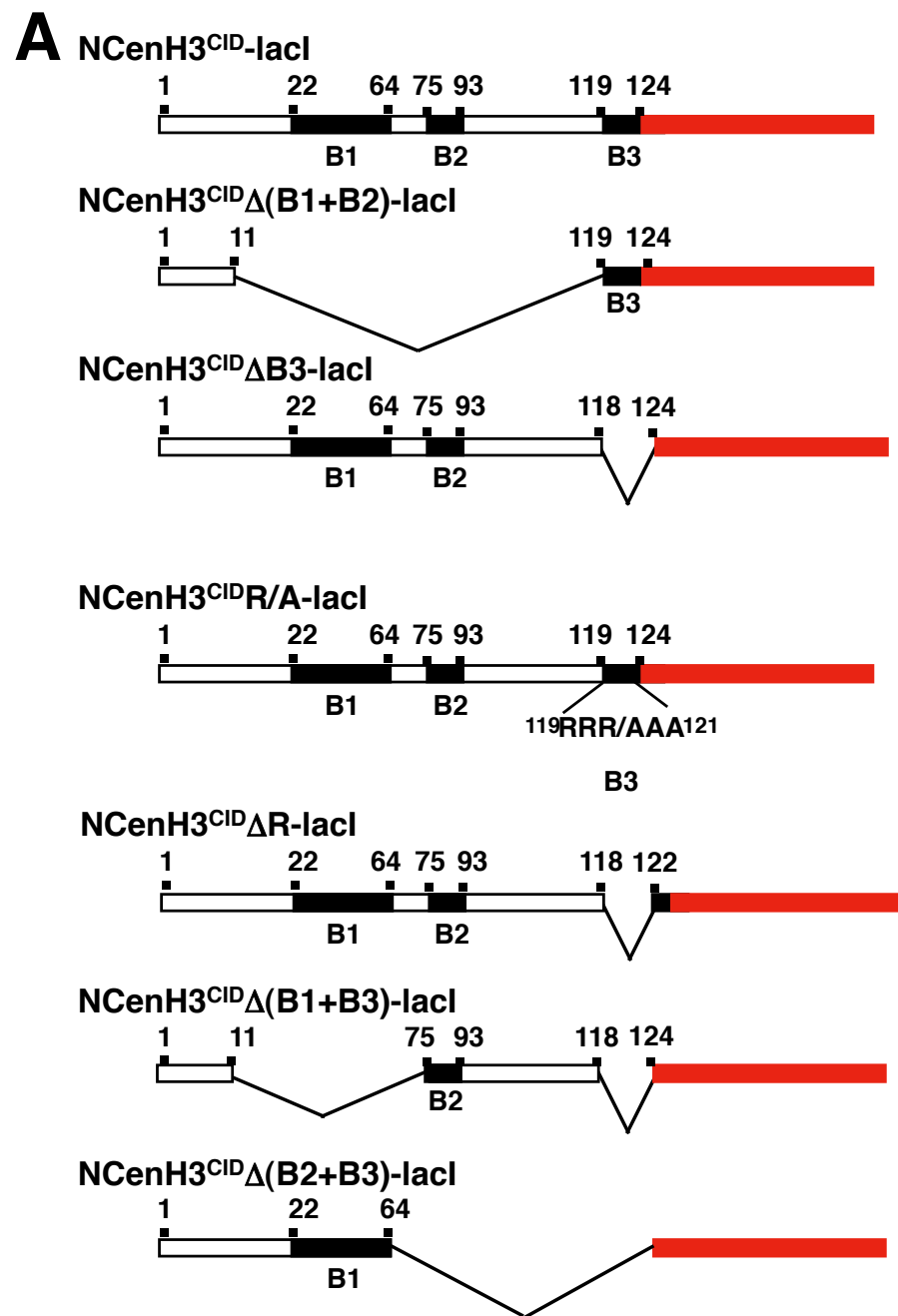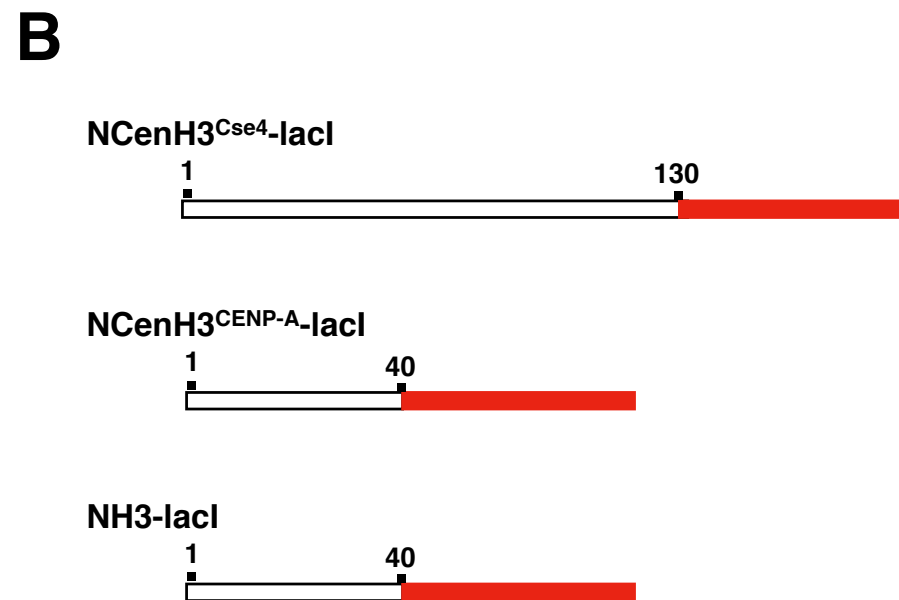

**FIGURE S4**

Supplement: Figure S4 — Constructs used in these experiments. Schematic representation of fused proteins used in these experiments. The position of motives B1, B2 and B3 is indicated. Numbers correspond to amino acid positions on the corresponding sequences. DNA-binding domain of lacI is indicated in red. (0.02 MB PDF) [file pone.0013747.s004.pdf]

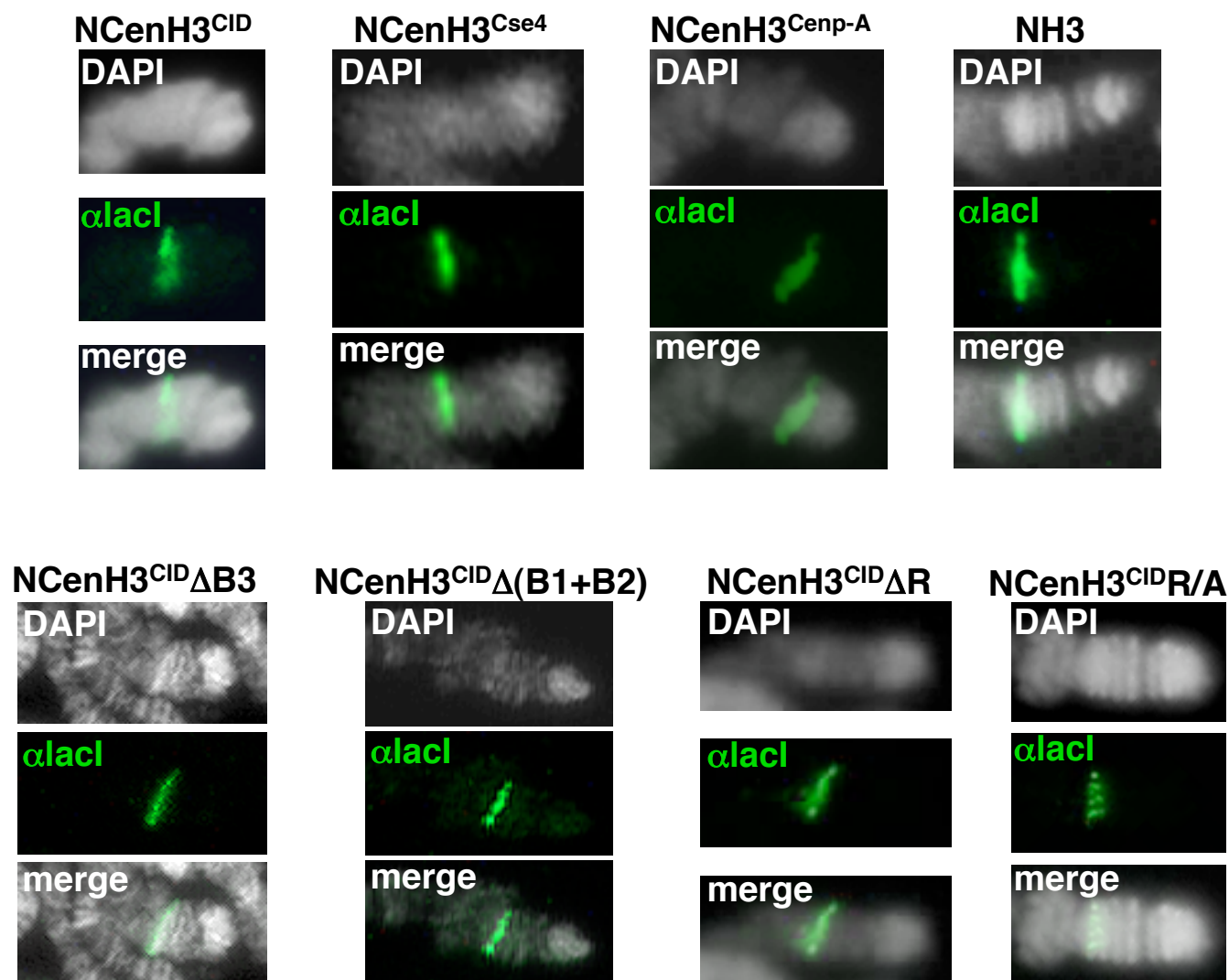

**FIGURE S5**

Supplement: Figure S5 — Constructs used in these experiments target the reporter construct in polytene chromosomes. Localisation of the indicated constructs was determined in polytene chromosomes by immunostaining with αlacI (green). DNA was stained with DAPI. (0.15 MB PDF) [file pone.0013747.s005.pdf]
